# PD-1-Positive Tumor-Associated Macrophages Define Poor Clinical Outcomes in Patients With Muscle Invasive Bladder Cancer Through Potential CD68/PD-1 Complex Interactions

**DOI:** 10.3389/fonc.2021.679928

**Published:** 2021-05-17

**Authors:** Li-Ren Jiang, Ning Zhang, Si-Teng Chen, Jin He, Yong-Hua Liu, Ya-Qin Han, Xiao-Qin Shi, Ji-Ji Yang, Dong-Yun Mu, Guo-Hui Fu, Feng Gao

**Affiliations:** ^1^ Pathology Center, Shanghai General Hospital, Shanghai Jiao Tong University School of Medicine, Shanghai, China; ^2^ Department of Urology, Ruijin Hospital, Shanghai Jiao Tong University School of Medicine, Shanghai, China; ^3^ Department of Urology, Shanghai General Hospital, Shanghai Jiao Tong University School of Medicine, Shanghai, China; ^4^ Department of Pathology, Zunyi Medical and Pharmaceutical College, Zunyi, China; ^5^ Key Laboratory of Cell Differentiation and Apoptosis of Chinese Ministry of Education, Institutes of Medical Sciences, Shanghai Jiao Tong University School of Medicine, Shanghai, China; ^6^ Shanghai Key Laboratory of Gastric Neoplasms, Shanghai Institute of Digestive Surgery, Ruijin Hospital, Shanghai Jiao Tong University School of Medicine, Shanghai, China

**Keywords:** PD-1, CD68, tumor-associated macrophages, muscle-invasive bladder cancer, prognosis

## Abstract

Tumor-associated macrophages (TAMs) regulate tumor immunity. Previous studies have shown that the programmed cell death protein 1 (PD-1)-positive TAMs have an M2 macrophage phenotype. CD68 is a biomarker of TAMs and is considered to be a poor prognostic marker of several malignancies. Our results show that PD-1-positive TAMs can be a negative survival indicator in patients with muscle-invasive bladder cancer (MIBC), and that the mechanistic effects could result due to a combination of PD-1 and CD68 activity. We analyzed 22 immune cell types using data from 402 patients with MIBC from the TCGA database, and found that a high immune score and M2 TAMs were strongly associated with poor clinical outcomes in patients with MIBC. Further, we analyzed resected samples from 120 patients with MIBC and found that individuals with PD-1-positive TAMs showed a reduction in 5-year overall survival and disease-free survival. Additionally, PD-1-positive TAMs showed a significant association with higher programmed death-ligand 1 (PD-L1) expression, the Ki67 index, the pT stage and fewer CD8-positive T cells. Through the co-immunoprecipitation (co-IP) assay of THP-1 derived macrophages, we found that CD68 can bind to PD-1. The binding of CD68 and PD-1 can induce M2 polarization of THP-1 derived macrophages and promote cancer growth. The anti-CD68 treatment combined with peripheral blood mononuclear cells (PBMC) showed obvious synergy effects on inhibiting the proliferation of T24 cells. Together, these results indicate for the first time that CD68/PD-1 may be a novel target for the prognosis of patients with MIBC.

## Introduction

Bladder cancer is the most common cancer of the urinary system that results in malignancies, and the resulting tumor types can be classified into non-muscle invasive bladder cancer (NMIBC) and muscle-invasive bladder cancer (MIBC) (1–3). Cisplatin is a first-line drug for the treatment of metastatic MIBC (4, 5). At present, there are no approved second-line drugs for patients with MIBC who show a relapse after cisplatin treatment; however, immunotherapy-related drugs can fill this gap (6).

In recent years, the use of PD-1/PD-L1 inhibitors has set a new paradigm for the treatment of patients with metastatic bladder cancer, and who are ineligible and resistant for cisplatin-based therapy (7). Nevertheless, the overall response rates are still improvable (5, 7). Further, ~10% of patients with bladder cancer treated with PD-1/PD-L1 inhibitors show rapid progression of the disease, known as hyper-progressive disease (8). Therefore, the mechanisms underlying the effects of these immunotherapeutic inhibitors are unclear and require further investigation.

PD-1 is an immunological checkpoint receptor which is upregulated in activated T cells and is important for inducing immune tolerance (9, 10). PD-L1 promotes the escape of tumor cells from the immune system (9, 10). Under normal conditions, PD-1 and PD-L1 are necessary for the maintenance of self-tolerance and for preventing T-cell-mediated immune stimulation (11, 12). However, cancer cells can acquire the property of immune escape through the expression of PD-L1 (13).

Tumor-associated macrophages (TAMs) show phenotypic plasticity and can be classified based on the M1 and M2 phenotypes (14, 15). The M1 macrophages are responsible for the removal of senescent/apoptotic cells and of foreign/pathogenic substances through phagocytosis, and are involved in the process of wound healing and tissue repair (16). The M2 macrophages are mainly involved in angiogenesis, wound healing, chronic infection, and in promoting the genesis of tumors and metastasis (17, 18). Recent studies have found that TAMs also express PD-1. These PD-1-positive TAMs exhibit M2 phenotypic characteristics and can promote tumor proliferation by suppressing tumor immunity (19).

CD68 is considered to be a valuable immunochemical marker which is used to identify monocytes/macrophages (20). Human CD68 belongs to the lysosomal-associated membrane protein (LAMP) family and is located in the lysosomal membrane though it can rapidly shuttle to the cell surface (20, 21). Additionally, CD68 is a receptor for the malaria sporozoite during liver infection (20, 22). However, the role of CD68 in tumor-induced immune suppression remains unclear (20). The expression of CD68 in both TAMs and tumor cells is related to poor clinical outcomes in various types of cancers (23).

In this study, we aimed to identify the association between M2 and PD-1-positive TAMs and clinical outcomes in patients with MIBC. Further, we hypothesized that PD-1-positive TAMs may potentially be regulated by the CD68 and PD-1 complex.

## Materials and Methods

### Patient Samples and Ethics Statement

The experimental protocol was designed according to the ethical guidelines of the Helsinki Declaration and was approved by the Human Ethics Committee of Shanghai General Hospital. Written informed consent was obtained from the individual or the guardians of the participants, as applicable.

A total of 339 patients with bladder cancer who were resected during 2007-2016 at the Shanghai General Hospital were enrolled in this study. Tissue samples were formalin-fixed and paraffin-embedded (FFPE). A total of 219 patients with bladder cancer were excluded from the study because of incomplete follow-up information, or because they were diagnosed as having NMIBC and non-urothelial carcinoma. Patients were excluded if they concurrently had HIV, other cancers, or autoimmune diseases. Further, the data from a total of 402 patients with MIBC from the Cancer Genome Atlas (TCGA) dataset was used in the study.

### Estimation of Immune Cell-Type Fractions

**Figure 1:**
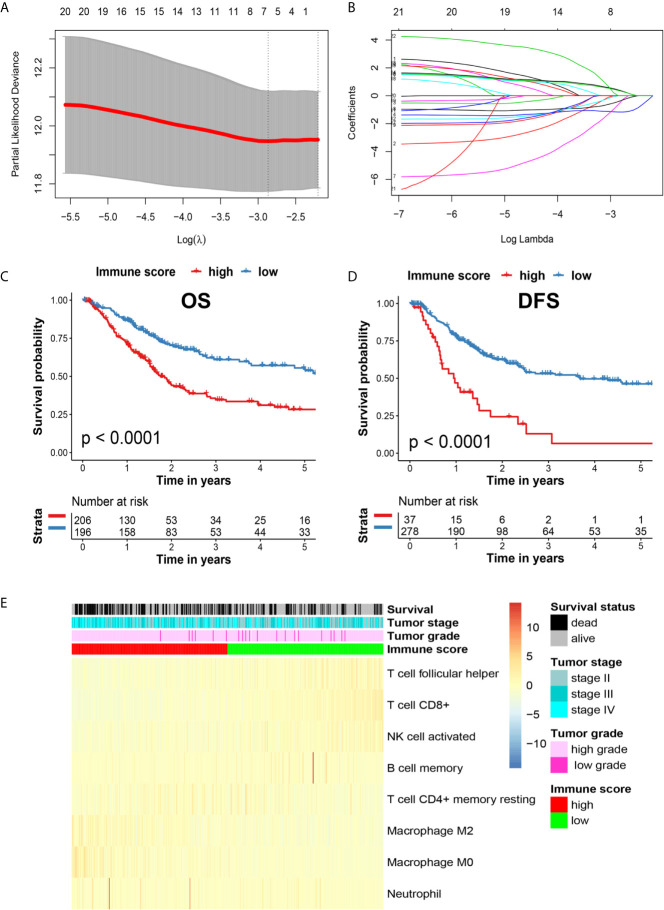
**(A)** Illustration of LASSO coefficient profiles of 22 immune cell types. **(B)** Cross-validation for the LASSO regression model. **(C)** Kaplan-Meier analysis of OS in high and low immune score patients with MIBC in the TCGA cohort. **(D)** Kaplan-Meier analysis of DFS in patients with high and low immune scores with MIBC in the TCGA cohort. **(E)** Heatmap of the related immune cells with high and low immune scores.

The RNA-Seq data and related clinical parameters were downloaded from the TCGA database, including gender, age, tumor grade, tumor stage, overall survival (OS) time, and disease-free survival (DFS) time. Next, we used CIBERSORT (https://cibersort.stanford.edu) to analyze the distribution of 22 tumor-infiltrating immune cell types using transcriptomic data from the TCGA database. All 22 immune cell types were analyzed by performing the least absolute shrinkage and selection operator (LASSO) method, and 8 immune cell types, including macrophage M0, macrophage M2, neutrophils, memory B cells, CD8^+^ T-cells, CD4^+^ T-cells, follicular T helper cells, and activated NK cells, were found to be significantly associated with the OS of patients with MIBC (**Figures 1A, B**). The immune score was calculated as follows: Macrophage M0 * 0.5958946 + macrophage M2 * 0.5450975 + neutrophils * 0.7150930 – memory B cells * 0.1278038 – CD8^+^ T-cells * 1.1224256 – CD4^+^ T-cells * 1.3433484 – follicular T helper cells * 0.6268039 – activated NK cells * 0.2084490.

### Immunohistochemistry (IHC)

FFPE samples were recovered from paraffin and incubated in a pressure oven for 30 min in EDTA (pH 9.0). Primary antibodies, mainly anti-PD-1 (Affinity; 1:100), anti-CD68 (Affinity; 1:100), anti-CD163 (Affinity; 1:100), anti-CD8 (Affinity; 1:100) and anti-PD-L1 (Dako; 1:50), were incubated with the samples overnight at 4°C. Next, the secondary antibodies were incubated with the samples for 60 min at room temperature. The sections on the slides were stained with 3, 3-diaminobenzidine and hematoxylin and visualized.

### Immunofluorescence Staining

FFPE samples were recovered from paraffin and incubated with EDTA (pH 9.0), followed by incubation with the mouse anti-PD-1 (Affinity; 1:100) or rabbit anti-CD68 (Abcam; 1:100) antibodies overnight at 4°C. Fluorescent secondary antibodies were labeled, and the nuclei were detected using DAPI staining. The slides were observed using an Olympus BX600 microscope and SPOT Flex camera.

### Cell Culture

THP-1 cell lines were cultured in RPMI 1640 medium (Gibco) supplemented with 10% fetal bovine serum (FBS, Gibco) and 1% penicillin-streptomycin (Gibco) at 37°C. The THP-1 cells were treated with propylene glycol methyl ether acetate (PMA, Sigma) at 37°C for 48 hours. The THP-1 derived macrophages and T24 bladder cancer cell lines were then cultured in RPMI 1640 medium (Gibco) supplemented with 10% FBS (Gibco) and 1% penicillin-streptomycin (Gibco) at 37°C. THP-1 derived macrophages were treated with 5µg/ml CD68 fusion protein (Abcam) and 10 µg/ml anti-CD68 antibody (Affinity) for 12 hours at 37°C, separately. PBMC were obtained from healthy donors and cultured in RPMI 1640 medium (Gibco) supplemented with 10% FBS (Gibco) and 1% penicillin-streptomycin (Gibco) at 37°C.

### Western Blot

THP-1 derived macrophages and T24 were lysed for 30 min at 44°C and centrifuged for 15 min at 4°C. The supernatant from the samples was collected and the total cell lysate protein concentrations were determined using the Bradford protein assay kit (Thermo Scientific). Next, equal amounts of protein were loaded, separated, and transferred to membranes, and these membrane-bound proteins were incubated overnight at 4°C with anti-PD-1 (Affinity; 1:500), anti-CD68 (Abcam; 1:1000), anti-PD-L1 (Dako; 1:500) and CD163 (Affinity; 1:1000) antibodies. The membrane was incubated with secondary antibodies for 40 min at room temperature. The protein bands were detected using an ECL kit (Merck Millipore).

### Co-Immunoprecipitation (co-IP)

THP-1 derived macrophages were lysed in RIPA buffer for 30 min at 4°C and then centrifuged at 12,000 rpm at 4°C for another 30 min. The lysates were incubated with protein A/G-agarose (Pierce) for 4 h at 4°C in a pre-clearing step. Specific antibodies were incubated overnight at 4°C, followed by an incubation of the antibody-treated lysate with protein-A/G agarose beads at 4°C for another 4 h. The beads were washed 6 times, mixed with loading buffer, and the eluted proteins were further examined by immunoblotting.

### Cell Viability Assay

The viability of co-culture of THP-1 derived macrophages, T24 cells and PBMCs after 12-hour treatment were assessed by the Cell Counting Kit-8 (CCK-8, MedChemExpress) assay. THP-1 derived macrophages, T24 cells and PBMCs were well mixed and seeded onto 96-well plates. Cells were treated with 5µg/ml CD68 fusion protein (Abcam), 10 µg/ml anti-CD68 antibody (Affinity) and 5µg/ml nivolumab (Bristol-Myers Squibb) for 12 hours at 37°C, separately. Afterwards, each well was added CCK-8 with a final concentration of 10% and incubated for 2 h at 37°C. The absorbance was measured at 450 nm by the microplate reader (Reagen).

### Molecular Docking

The X-ray structures of the IgV domain of PD-1 and the conserved LAMP-like domain of DC-LAMP of CD68 were downloaded from the protein data bank website (www.rcsb.orb). The X-ray structure of the LAMP-like domain of CD68 was modeled based on the X-ray structure from DC-LAMP by using the (PS) 2v2 program (24). Based on the X-ray structure of PD-1 and the predicted X-ray structure of the LAMP-like domain of CD68, we used the ClusPro web server to perform protein docking simulations.

### Statistical Analysis

Statistical analysis was performed using SPSS 20.0. The OS and DFS were analyzed *via* Kaplan–Meier analysis and the log-rank test. The prognostic significance of the clinicopathological parameters was analyzed using the chi-square test. The Spearman correlation analysis was used to analyze the correlation between CD68, PD-1, and PD-L1. The relationship of PD-1-positive TAMs CD8-positive T cells was analyzed by Student’s *t*-test. A *p-*value of <0.05 was considered to indicate a statistically significant difference in all the tests performed.

## Results

### The Presence of M2 TAMs Indicated Poor Clinical Outcomes in MIBC Patients

**Figure 2:**
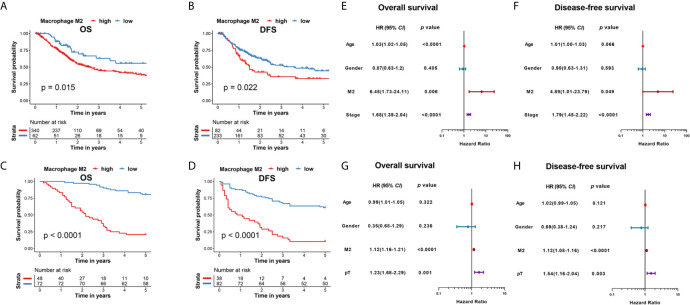
**(A)** Kaplan–Meier analysis of OS in patients with MIBC with M2 TAMs in the TCGA cohort. **(B)** Kaplan–Meier analysis of DFS in patients with MIBC with M2 TAMs in the TCGA cohort. **(C)** Kaplan–Meier analysis of OS in patients with MIBC with M2 TAMs in the Shanghai General Hospital cohort. **(D)** Kaplan–Meier analysis of DFS in patients with MIBC with M2 TAMs in the Shanghai General Hospital cohort. **(E)** Forest plot showing the hazard ratios with a 95% confidence interval for OS in the TCGA cohort. **(F)** Forest plot showing the hazard ratios with a 95% confidence interval for DFS in the TCGA cohort. **(G)** Forest plot showing the hazard ratios with a 95% confidence interval for OS in the Shanghai General Hospital cohort. **(H)** Forest plot showing the hazard ratios with a 95% confidence interval for DFS in the Shanghai General Hospital cohort.

We analyzed the presence of 22 immune cell types in patients with MIBC using data from the TCGA database *via* a LASSO analysis, and identified 8 survival-associated immune cell types. Further, we derived an immune score for predicting the prognosis of patients with MIBC (**Figures 1A, B**). According to the Kaplan-Meier analysis of the TCGA cohort, patients with MIBC having high immune scores were associated with a significantly reduced 5-year OS and DFS outcomes (*p* < 0.0001 and *p* < 0.0001, respectively; **Figures 1C, D**). The heatmap of the survival, tumor stage, tumor grade, immune score, and the profiles of different immune cells is shown in **Figure 1E**. Interestingly, M2 TAMs showed a correlation with high immune scores in the heatmap (**Figure 1E**). Therefore, we further analyzed the clinical outcomes for patients with MIBC showing the presence of M2 TAMs in the TCGA cohort. Patients with M2 TAMs showed significantly worse 5-year OS and DFS outcomes (*p*=0.015 and *p*=0.022, respectively; **Figures 2A, B**). Furthermore, high M2 TAMs were also associated with significantly worse 5-year OS and DFS outcomes in the Shanghai General Hospital cohort (p < 0.0001 and p < 0.0001, respectively; **Figures 2C, D**). The hazard ratio with a 95% confidence interval is shown in the forest plots (**Figures 2E–H**).

### PD-1-Positive TAMs Were Correlated With Reduced Survival in Patients With MIBC

**Figure 3:**
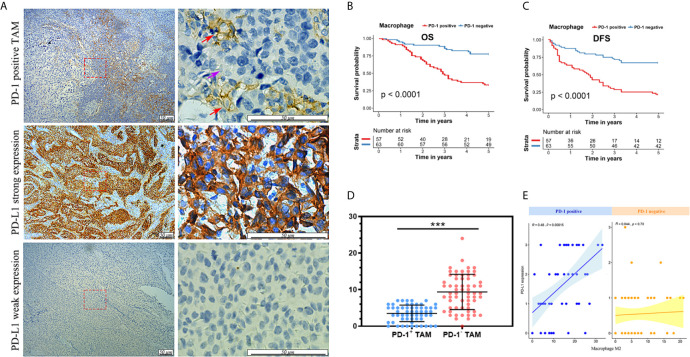
**(A)** Immunohistochemical staining of PD-1-positive TAMs (red arrow), PD-1-negative TAMs (purple arrow), strong PD-L1 and weak PD-L1 expression. **(B)** Kaplan-Meier analysis of OS in patients with MIBC with PD-1-positive TAMs in the Shanghai General Hospital cohort. **(C)** Kaplan-Meier analysis of DFS in patients with MIBC with PD-1-positive TAMs in the Shanghai General Hospital cohort. **(D)** PD-1-positive TAMs showed less CD8-postive T cells nearby. ***p < 0.001 (Student’s *t*-test). **(E)** The number of PD-1-positive TAMs showed a correlation with PD-L1 expression in the Shanghai General Hospital cohort.

As PD-1-positive TAMs exhibit M2 characteristics, we analyzed the clinicopathological features of PD-1-positive TAMs among patients with MIBC that were resected at the Shanghai General Hospital. Representative PD-1-positive TAMs and PD-L1 expression levels are shown in **Figure 3A**, and the patient characteristics are listed in **Table 1**. PD-1-positive TAMs were found in 47.5% of the 120 patients with MIBC. The median follow-up duration among patients with PD-1-positive and -negative TAM was 35 months and 60 months, respectively. Patients with MIBC having PD-1-positive TAMs were significantly associated with a decreased 5-year OS and DFS (*p* < 0.001 and *p* < 0.001, respectively; **Figures 3B, C**). The 5-year OS rate was estimated to be ~33.3% in patients with PD-1-positive TAMs, and ~77.78% in patients with PD-1-negative TAMs (**Figure 3B**). Additionally, the 5-year DFS rate was ~21.1% in patients with PD-1-positive TAM compared with ~66.7% in patients with PD-1-negative TAM (**Figure 3C**).

**Table 1:** Association of PD-1 positive and negative TAMs with clinicopathological parameters.

	PD1+TAM	PD1-TAM	*p*-value
**Number of patients**	57	63	
			
**Age**			0.338
** Median**	67	66	
			
**Gender**			0.806
** Male**	48	52	
** Female**	9	11	
			
**pT stage**			0.027
** pT2**	26	44	
** pT3**	16	10	
** pT4**	15	9	
			
**Histology**			<0.001
** Micropillar**	7	1	
** Nest**	32	47	
** Trabecular**	5	0	
** Papillar**	6	15	
** Undifferentiated**	7	0	
			
**PDL1**			<0.001
** 3+**	16	1	
** 2+**	13	2	
** 1+**	17	27	
**-**	11	33	
			
**Ki67**			0.003
** >=50%+**	28	10	
** <50%+**	29	53	
			
**Events**			
** Death**	38	14	
** Recurrence**	45	21	

Moreover, PD-1-positive TAMs were significantly correlated with pT stage clinicopathological features (*p* = 0.027, **Table 1**). Patients with PD-1-positive TAMs were found to exhibit higher pT stages than those without. PD-1-positive TAMs also showed significantly stronger PD-L1 expression (*p* < 0.001), a higher Ki67 index (*p* = 0.003), and worse pathological patterns (*p* < 0.001; **Table 1**). Interestingly, higher PD-L1 expression levels also resulted in poor prognosis of patients with MIBC (data not shown).

We investigated the response to cisplatin-based neoadjuvant chemotherapy in patients with PD-1-positive and -negative TAM phenotypes. Patients with MIBC who were administered neoadjuvant chemotherapy in the pT2 stage showed a better prognosis. However, the presence of PD-1-positive and -negative TAM did not improve the response to neoadjuvant chemotherapy (*p* > 0.05, data not shown). Patients with a PD-1-positive TAM phenotype showed a comparatively inferior response to neoadjuvant chemotherapy, which was similar to the 5-year OS and DFS outcomes in patients with PD-1-positive TAMs.

Intriguingly, PD-1-positive TAMs showed relevance to bladder cancer related immune response. Based on 120 MIBC patients from Shanghai General Hospital, we found that CD8-positve T cells were comparatively fewer around PD-1-positive TAMs (*p* < 0.001; **Figure 3D**), indicating PD-1-positive TAMs could be involved in bladder cancer immune response. In addition, the number of PD-1-positive TAMs showed positive relevance to the PD-L1 expression of bladder cancer cells (*R* = 0.48, *p* < 0.001; **Figure 3E**).

### The Interaction of CD68 and PD-1 Induced TAMs to M2 Polarization

**Figure 4:**
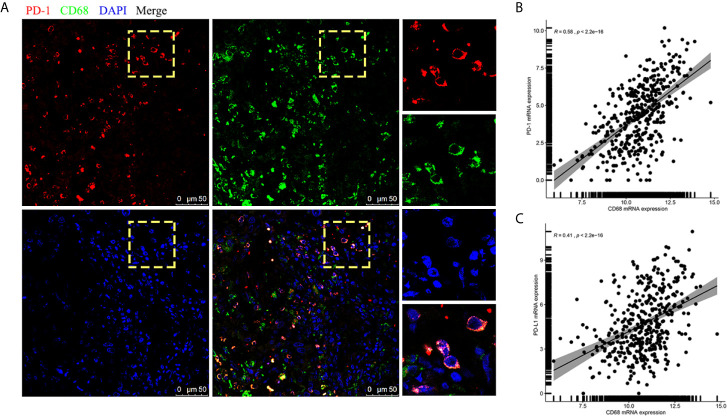
**(A)** Immunofluorescence staining confirmation for the appearance of PD-1-positive TAMs. **(B)** mRNA expression of CD68 showed a correlation with PD-1 expression in the TCGA cohort. **(C)** mRNA expression of CD68 showed a correlation with PD-L1 expression in the TCGA cohort.

**Figure 5:**
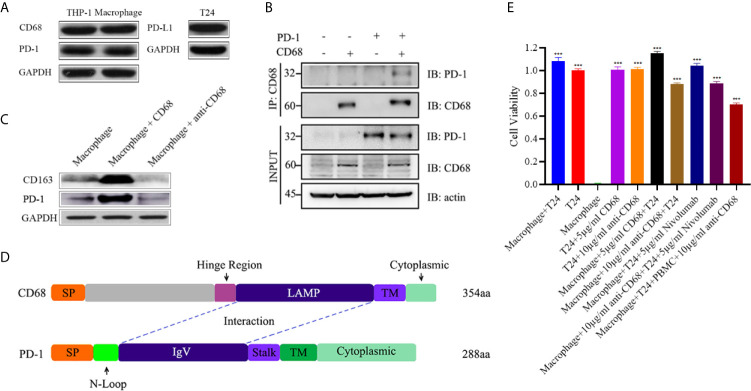
**(A)** Both THP-1 cells and THP-1 derived macrophages expressed PD-1 and CD68. T24 cells expressed PD-L1. **(B)** A co-IP assay showing the binding between CD68 and PD-1, hinting at the possibility of a CD68 and PD-1 interaction. **(C)** The binding of CD68 and PD-1 promoted THP-1 derived macrophages to M2 polarization whereas the blockage can reverse the process. **(D)** Molecular docking showing the interactions of CD68 and PD-1 through possible LAMP-like and IgV domains. SP indicates signal peptide and TM indicates transmembrane domain. **(E)** The cell viability assays of T24, THP-1 derived macrophages and PBMC co-culture experiments. T24 cell group and THP-1 derived macrophage group were the control groups. ***p < 0.001 (Student’s *t*-test).

Interestingly, when we further analyzed the existence of PD-1-positive TAMs using immunofluorescence staining on FFPE samples, CD68 and PD-1 tended to be expressed synchronously in TAMs (**Figure 4A**). Hence, we conducted an analysis for the correlation of CD68 mRNA expression levels with the PD-1 and PD-L1 mRNA expression levels in the TCGA cohort and found that the expression of CD68 and that of PD-1 and PD-L1 was correlated (*R* = 0.58 and *R* = 0.41, respectively; *p* < 0.001 and *p* < 0.001, respectively; **Figures 4B, C**). Towards this, we conducted a co-immunoprecipitation assay in THP-1 derived macrophages, which expressed both CD68 and PD-1, to test for a possible interaction between the two proteins (**Figure 5A**). Our results showed that the anti-CD68 antibody could precipitate both CD68 and PD-1, indicating that CD68 could bind to PD-1 *in vitro* (**Figure 5B**).

Based on the results of the co-immunoprecipitation assay, we next explored the function of CD68/PD-1 interaction. After 12-hour treatment of CD68 and anti-CD68 on the THP-1 derived macrophages, we found that the interaction of CD68 and PD-1 can increase the CD163 levels, indicating the M2 polarization of THP-1 derived macrophages (**Figure 5C**). In addition, the block of CD68 to PD-1 can reverse the M2 polarization (**Figure 5C**).

Furthermore, the potential binding of CD68 and PD-1 were estimated *in silico*. We identified the functionally conserved LAMP-like domain of CD68 and the IgV domain of PD-1, and analyzed the potential binding between these two domains through molecular docking (**Figure 5D**).

### Anti-CD68 With PD-1-Positive TAMs Existence Can Mediate Antitumor Effects

To determine whether CD68/PD-1 interaction can interfere the bladder cancer growth, T24 cells, expressing PD-L1, were co-cultured with THP-1 derived macrophages and treated with 5µg/ml CD68, 10µg/ml anti-CD68 and 5µg/ml Nivolumab, separately (**Figures 5A, E**). After 12-hour treatment, 5µg/ml CD68 and 10µg/ml anti-CD68 alone did not show obvious changes in T24 cell growth (**Figure 5E**). When together with THP-1 derived macrophages, 5µg/ml CD68 promoted T24 cell proliferation and 10µg/ml anti-CD68 inhibited T24 cell growth (**Figure 5E**). 5µg/ml Nivolumab also showed its inhibition on T24 cell growth but the synergy effects of anti-CD68 and Nivolumab were not obvious (**Figure 5E**). Moreover, the co-culture of PBMCs, THP-1 derived macrophages and T24 cells suggested that the anti-CD68 treatment combined with PBMCs showed obvious synergy effects on inhibiting the proliferation of T24 cells, indicating anti-CD68- mediated antitumor effect was T-cell dependent (**Figure 5E**).

## Discussion

The clinical application of PD-1/PD-L1 related immunotherapies implies the potential immunogenicity of bladder cancer (5, 25). However, PD-1/PD-L1 related immunotherapy for bladder cancer, especially for patients with MIBC, is still improvable (5), as these are effective in only a certain proportion of patients with bladder cancer (26). Some patients with MIBC undergoing PD-1 related therapies have progressed to develop hyper-progressive disease (8). Therefore, we aimed to identify alternate immune therapy targets in patients with MIBC. This study is the first to demonstrate that CD68 can bind to PD-1, which may provide a new target for future immunotherapeutic strategies.

Further, the expression of CD68 and PD-1, which indicates the existence of PD-1-positive TAMs, may be used as a predictive biomarker for individuals with MIBC. Studies have shown that PD-1-positive TAMs show characteristics of the M2 phenotype, and are correlated with tumor progression *in vitro*, and *in vivo* (19, 27). In this study, we showed that M2 TAMs were a negative survival indicator for patients with MIBC in the TCGA cohort. Next, we reported that the presence of PD-1-positive TAMs in patients with MIBC indicated a reduced 5-year OS and DFS. PD-1-positive TAMs were also correlated with higher undifferentiated histological patterns, which indicate worse clinical outcomes (5). Further, PD-1-positive TAMs were also linked to deeper invasion of MIBC, indicating a higher pT stage. With a higher Ki67 expression in the surrounding bladder cancer cells, the presence of PD-1-positive TAMs also suggested greater tumor proliferation. Together, these findings suggest that PD-1 and CD68 can be regarded as negative survival indicators for patients with MIBC.

Although CD68 has found use in clinical settings as the biomarker for macrophages for decades, the true function of CD68 in tumor immune regulation remains unclear (20). The focus on the role of PD-1/PD-L1 in tumor immunity has involved T cells for a long time (19). However, as PD-1-positive TAMs can directly affect tumor cell proliferation, we explored the mechanisms underlying the upstream regulation of PD-1/PD-L1 in TAMs (19). Using bioinformatics analysis, we found that upon binding to PD-1, CD68 was expected to reduce the van der Waals interaction energy and electrostatic energy. This implied that the CD68 and PD-1 complex might be stabilized through the binding of LAMP-like and IgV domains, enhancing the tumor immune inhibition function of PD-1. Moreover, CD68 is a lysosomal membrane protein that can rapidly shuttle to the cell surface (20, 21). Therefore, we hypothesized that CD68 receives signals from upstream factors and transfers to the cell surface and binds to PD-1, which initiates the regulation of the PD-1/PD-L1 pathway in TAMs. We also estimated the relationship of CD68 and PD-1/PD-L1 complex by using the (PS)2v2 program. The X-ray structure of the PD-1/PD-L1 complex was downloaded from the protein data bank website. Although the results from the molecular docking analysis indicate that CD68 may not directly affect the PD-1/PD-L1 complex, CD68, PD-1, and PD-L1 may form a complex. In additon, through *in vitro* experiments, we found that CD68/PD-1 interaction can induce THP-1 derived macrophages to M2 polarization and the block of CD68/PD-1 interaction can inhibit T24 cell growth. The anti-CD68-mediated antitumor effect was T-cell dependent and anti-CD68-induced antitumor T-cell immunity was macrophage dependent.

Anti-PD-1 immunotherapy is a promising future therapy for patients with MIBC (5). The resection of the whole bladder is an agonizing process for patients with MIBC, and in practice, it remains an obstacle for future anti-PD-1 based immunotherapy (5). Therefore, the potential binding of CD68/PD-1 could provide a new therapeutic target. However, a detailed functional analysis of CD68 and PD-1 in the context of bladder cancer requires further investigation. The results from our study showing the formation of a CD68/PD-1 complex may prove to be valuable in the field of MIBC tumor immunity research.

To conclude, M2 and PD-1-positive TAMs are associated with poor clinical outcomes in MIBC patients. We found that PD-1-positive TAMs can be potentially regulated by the CD68 and PD-1 complex, and that the interaction of CD68 and PD-1 indicated poor clinical outcomes in patients with MIBC.

## Data Availability Statement

The original contributions presented in the study are included in the article/supplementary material. Further inquiries can be directed to the corresponding authors.

## Ethics Statement

The studies involving human participants were reviewed and approved by The Human Ethics Committee of Shanghai General Hospital. The patients/participants provided their written informed consent to participate in this study.

## Author Contributions

L-RJ conceived the idea for the study, conducted cell cultures, cell viability assays, co-IP, and immunofluorescence staining, and was a major contributor in writing the manuscript. NZ obtained fresh samples from patients with MIBC, analyzed the patient data, and was the second leading contributor for manuscript preparation. S-TC conducted the bioinformatics analysis and was a major contributor for revising the manuscript. JH performed the IHC staining, Y-HL performed the western blot assays, and Y-QH collected and prepared the FFPE samples. X-QS gathered the patients’ medical histories and pathological diagnoses. J-JY and D-YM helped culture the cells and gathered all the patient consent forms as well as the ethics statements. G-HF provided suggestions on the project throughout the study, joined the interpretation of the results, gave important assistance in the bioinformatics analysis, and was the major contributor for manuscript revision. FG developed the idea for the study, contributed the central idea, funded the research, and was the second leading contributor for manuscript revision. All authors contributed to the article and approved the submitted version.

## Funding

This work was supported by the Shanghai Municipal Key Clinical Specialty (shslczdzk01303).

## Conflict of Interest

The authors declare that the research was conducted in the absence of any commercial or financial relationships that could be construed as a potential conflict of interest.

## References

[1] Chen W Zheng R Baade PD Zhang S Zeng H Bray F. Cancer Statistics in China, 2015. CA Cancer J Clin (2016) 66:115–32. 10.3322/caac.21338 26808342

[2] Schneider AK Chevalier MF Derre L. The Multifaceted Immune Regulation of Bladder Cancer. Nat Rev Urol (2019) 16:613–30. 10.1038/s41585-019-0226-y 31501534

[3] Flaig TW Spiess PE Agarwal N Bangs R Boorjian SA Buyyounouski MK. NCCN Guidelines Insights: Bladder Cancer, Version 5.2018. J Natl Compr Cancer Netw (2018) 16:1041–53. 10.6004/jnccn.2018.0072 30181416

[4] Butt SUR Malik L. Role of Immunotherapy in Bladder Cancer: Past, Present and Future. Cancer Chemother Pharmacol (2018) 81:629–45. 10.1007/s00280-018-3518-7 29368051

[5] Boegemann M Aydin AM Bagrodia A Krabbe LM. Prospects and Progress of Immunotherapy for Bladder Cancer. Expert Opin Biol Ther (2017) 17:1417–31. 10.1080/14712598.2017.1366445 28832261

[6] Song D Powles T Shi L Zhang L Ingersoll MA Lu YZ. Bladder Cancer, a Unique Model to Understand Cancer Immunity and Develop Immunotherapy Approaches. J Pathol (2019) 249:151–65. 10.1002/path.5306 PMC6790662 31102277

[7] Tan WP Tan WS Inman BA. Pd-L1/Pd-1 Biomarker for Metastatic Urothelial Cancer That Progress Post-Platinum Therapy: A Systematic Review and Meta-Analysis. Bladder Cancer (2019) 5:211–23. 10.3233/BLC-190238 PMC6919639 31867425

[8] Hwang I Park I Yoon SK Lee JL. Hyperprogressive Disease in Patients With Urothelial Carcinoma or Renal Cell Carcinoma Treated With Pd-1/Pd-L1 Inhibitors. Clin Genitourin Cancer (2020) 18:e122–33. 10.1016/j.clgc.2019.09.009 31837940

[9] Wang F Li B Wei W Zhao Y Wang L Zhang P. Tumor-Derived Exosomes Induce PD1(+) Macrophage Population in Human Gastric Cancer That Promotes Disease Progression. Oncogenesis (2018) 7:41. 10.1038/s41389-018-0049-3 29799520 PMC5968036

[10] Bardhan K Anagnostou T Boussiotis VA. The PD1:PD-L1/2 Pathway From Discovery to Clinical Implementation. Front Immunol (2016) 7:550. 10.3389/fimmu.2016.00550 28018338 PMC5149523

[11] Hato T Goyal L Greten TF Duda DG Zhu AX. Immune Checkpoint Blockade in Hepatocellular Carcinoma: Current Progress and Future Directions. Hepatology (2014) 60:1776–82. 10.1002/hep.27246 PMC4211962 24912948

[12] Pardoll DM. The Blockade of Immune Checkpoints in Cancer Immunotherapy. Nat Rev Cancer (2012) 12:252–64. 10.1038/nrc3239 PMC4856023 22437870

[13] Mocan T Sparchez Z Craciun R Bora CN Leucuta DC. Programmed Cell Death Protein-1 (PD-1)/programmed Death-Ligand-1 (PD-L1) Axis in Hepatocellular Carcinoma: Prognostic and Therapeutic Perspectives. Clin Transl Oncol (2019) 21:702–12. 10.1007/s12094-018-1975-4 30387047

[14] Sha H Zhang D Zhang Y Wen Y Wang Y. ATF3 Promotes Migration and M1/M2 Polarization of Macrophages by Activating Tenascinc Via Wnt/betacatenin Pathway. Mol Med Rep (2017) 16:3641–7. 10.3892/mmr.2017.6992 28714032

[15] Wang Y Smith W Hao D He B Kong L. M1 and M2 Macrophage Polarization and Potentially Therapeutic Naturally Occurring Compounds. Int Immunopharmacol (2019) 70:459–66. 10.1016/j.intimp.2019.02.050 30861466

[16] Lewis CE Harney AS Pollard JW. The Multifaceted Role of Perivascular Macrophages in Tumors. Cancer Cell (2016) 30:18–25. 10.1016/j.ccell.2016.05.017 27411586 PMC5024543

[17] Liu M Tong Z Ding C Luo F Wu S Wu C. Transcription Factor c-Maf is a Checkpoint That Programs Macrophages in Lung Cancer. J Clin Invest (2020) 130:2081–96. 10.1172/JCI131335 PMC7108920 31945018

[18] Zhou Y Yoshida S Kubo Y Yoshimura T Kobayashi Y Nakama T. Different Distributions of M1 and M2 Macrophages in a Mouse Model of Laser-Induced Choroidal Neovascularization. Mol Med Rep (2017) 15:3949–56. 10.3892/mmr.2017.6491 PMC5436148 28440413

[19] Gordon SR Maute RL Dulken BW Hutter G George BM McCracken MN. PD-1 Expression by Tumour-Associated Macrophages Inhibits Phagocytosis and Tumour Immunity. Nature (2017) 545:495–9. 10.1038/nature22396 PMC5931375 28514441

[20] Chistiakov DA Killingsworth MC Myasoedova VA Orekhov AN Bobryshev YV. CD68/Macrosialin: Not Just a Histochemical Marker. Lab Invest (2017) 97:4–13. 10.1038/labinvest.2016.116 27869795

[21] Holness CL da Silva RP Fawcett J Gordon S Simmons DL. Macrosialin, a Mouse Macrophage-Restricted Glycoprotein, is a Member of the Lamp/Lgp Family. J Biol Chem (1993) 268:9661–6. 10.1016/S0021-9258(18)98400-0 8486654

[22] Cha SJ Park P Srinivasan P Schindler CW van Rooijen N Stins M. CD68 Acts as a Major Gateway for Malaria Sporozoite Liver Infection. J Exp Med (2015) 212:1391–403. 10.1084/jem.20110575 PMC4548058 26216124

[23] Alessandrini F Pezze L Ciribilli Y. Lamps: Shedding Light on Cancer Biology. Semin Oncol (2017) 44:239–53. 10.1053/j.seminoncol.2017.10.013 29526252

[24] Chen CC Hwang JK Yang JM. (Ps)2-v2: Template-Based Protein Structure Prediction Server. BMC Bioinf (2009) 10:366. 10.1186/1471-2105-10-366 PMC2775752 19878598

[25] Wang B Wu S Zeng H Liu Z Dong W He W. He, W CD103+ Tumor Infiltrating Lymphocytes Predict a Favorable Prognosis in Urothelial Cell Carcinoma of the Bladder. J Urol (2015) 194:556–62. 10.1016/j.juro.2015.02.2941 25752441

[26] Hussain SA Birtle A Crabb S Huddart R Small D Summerhayes M. From Clinical Trials to Real-Life Clinical Practice: The Role of Immunotherapy With PD-1/PD-L1 Inhibitors in Advanced Urothelial Carcinoma. Eur Urol Oncol (2018) 1:486–500. 10.1016/j.euo.2018.05.011 31158093

[27] Li B Song TN Wang FR Yin C Li Z Lin JP. Tumor-Derived Exosomal HMGB1 Promotes Esophageal Squamous Cell Carcinoma Progression Through Inducing PD1(+) TAM Expansion. Oncogenesis (2019) 8:17. 10.1038/s41389-019-0126-2 30796203 PMC6386749

